# Vacuum ultraviolet photofragmentation of octadecane: photoionization mass spectrometric and theoretical investigation

**DOI:** 10.1007/s13203-015-0119-9

**Published:** 2015-07-07

**Authors:** Jing Xu, Pengpeng Sang, Lianming Zhao, Wenyue Guo, Fei Qi, Wei Xing, Zifeng Yan

**Affiliations:** 1 College of Science, China University of Petroleum, Qingdao, 266580 Shandong People’s Republic of China; 2 National Synchrotron Radiation Laboratory, University of Science and Technology of China, Hefei, 230029 Anhui People’s Republic of China; 3 State Key Laboratory of Heavy Oil Processing, Key Laboratory of Catalysis, China University of Petroleum, Qingdao, 266580 People’s Republic of China

**Keywords:** Synchrotron vacuum ultraviolet, Photoionization, Alkanes, Mass spectrometry

## Abstract

The photoionization and fragmentation of octadecane were investigated with infrared laser desorption/tunable synchrotron vacuum ultraviolet (VUV) photoionization mass spectrometry (IRLD/VUV PIMS) and theoretical calculations. Mass spectra of octadecane were measured at various photon energies. The fragment ions were gradually detected with the increase of photon energy. The main fragment ions were assigned to radical ions (C_*n*_H_2*n*+1_^+^, *n* = 4–11) and alkene ions (C_*n*_H_2*n*_^+^, *n* = 5–10). The ionization energy of the precursor and appearance energy of ionic fragments were obtained by measuring the photoionization efficiency spectrum. Possible formation pathways of the fragment ions were discussed with the help of density functional theory calculations.

## Introduction

With the increasing demand for energy and ongoing depletion of light oil resources, high-efficient use of heavy oils is becoming more and more attractive. To explore the extreme refinement of heavy oils, it is necessary to deeply understand their compositions and structures [1, 2]. It is known that petroleum residues can be divided into saturates, aromatics, resins, and asphaltenes (SARA) according to the molecular polarity and solubility. In general, saturates are primarily consist of saturated alkanes and cycloalkanes. On the other hand, the pyrolysis of crude oil is considered as one of major sources of natural gas. In crude oil, one of the main components is alkanes. Therefore, study of alkane cracking is important to understand the genesis of natural gas. As is well known, octadecane is a prototype of the class of *n*-alkanes, and thus it is very interesting to study its property and decomposition mechanism.

In recent decades, various techniques have been applied to analyze petroleum [3–9]. These methods include fluorescent indicator adsorption [4], infrared (IR)/Fourier-transform infrared (FTIR) spectroscopy [7], nuclear magnetic resonance (NMR) spectroscopy [8], mass spectrometry (MS) [3, 6], gas chromatography (GC) [9], and so on. Among them, MS always shows the predominance in the analysis of petroleum due to its accuracy and high speed. Recently, as a powerful detection tool, photoionization mass spectrometry (PIMS) has been used extensively for analyzing organic analytes and studying combustion [10–12]. However, experimental measurements of photoionization for alkanes are scarce. Kameta et al. measured the photoionization and dissociation properties of methane, ethane, propane, cyclopropane, and *n*-butane using a double ionization chamber combined with synchrotron radiation [13]. Steiner et al. reported the photoionization and subsequent dissociation of all saturated paraffins from C_2_ to C_6_, plus *n*-heptane and *n*-octane using a mass spectrometer combined with a Seya–Namioka monochromator [14]. Schoen measured the ionization and ion-fragmentation cross sections of ethane, propane, *n*-butane, *n*-pentane, cyclopropane, etc., under vacuum ultraviolet radiation [15]. The photoionization cross sections of *n*-pentane, *n*-hexane, *n*-heptane, *n*-octane, *n*-nonane, and *n*-decane were measured exclusively at 10.5 eV by Adam and Zimmermann [16]. The near-threshold photoionization cross sections for methane, ethane, propane, *n*-butane, cyclopropane, and methylcyclopentane were measured by Cool and co-workers [17] using PIMS combined with vacuum ultraviolet (VUV) synchrotron radiation. Recently, the photoionization and dissociative photoionization cross sections of eleven *n*-alkanes, three cyclo-alkanes, and iso-octane were measured by Zhou et al., utilizing tunable synchrotron VUV photoionization and molecular-beam mass spectrometry [18]. Although photoionization properties are available for some small alkanes, the photoionization investigations of large alkanes are very sparse.

In this work, we investigated the photoionization and fragmentation behavior of octadecane using infrared laser desorption/tunable VUV PIMS (IRLD/VUV PIMS) and theoretical calculations. The photoionization mass spectra of octadecane were obtained at different photon energies. The ionization energy (IE) of octadecane and appearance energy (AE) of fragments were obtained by measuring the photoionization efficiency (PIE) spectrum. Furthermore, the major dissociation pathways to form radical C_*n*_H_2*n*+1_^+^ (*n* = 4–11) and alkene C_*n*_H_2*n*_^+^ (*n* = 5–10) fragments were presented on the basis of density functional theory calculations.

## Experimental and theoretical methods

### Experimental method

The experiment was completed at the National Synchrotron Radiation Laboratory, Hefei, China. The IR LD/VUV PIMS setup was described in detail in previous publications [19, 20]. Briefly, the instrument used a Nd:YAG laser beam (Surelite I-20; Continuum, Santa Clara, CA, USA; wavelength 1064 nm, repetition rate 10 Hz) for desorption of samples mounted on a stainless steel substrate. To generate the plume of intact neutral molecules, the laser power for desorption was controlled at about 6 mJ/pulse. The desorbed neutral molecules in the gas phase were ionized by the crossed synchrotron VUV light, and the generated ions were detected by a home-made reflection time-of-flight (RTOF) mass spectrometer. The ion signals were amplified by a preamplifier (VT120C, EG & G, ORTEC, U.S.A.) and recorded by a P7888 multiscaler (FAST Comtec, Germany). Time delay between the laser and the pulse of repeller field of RTOF is 150 μs, which was controlled by a homemade pulse/delay generator.

Synchrotron VUV radiation from an undulator beamline of 800 MeV electron storage ring of the NSRL was monochromatized by a 1 m Seya–Namioka monochromator with a laminar grating (1500 grooves mm^−1^, Horiba Jobin–Yvon, France). The grating covered the photon energy range from 7.8 to 24 eV with the energy resolution (*E*/Δ*E*) of about 1000. The monochromator was calibrated with known IEs of inert gases. A gas filter filled with neon or argon was used to eliminate higher order harmonic radiation. The average photon flux was measured to be 1 × 10^13^ photons/s. A silicon photodiode (SXUV-100, International Radiation Detectors Inc., U.S.A.) was used to monitor the photon flux for normalizing ion signals.

### Computational method

All the theoretical calculations were performed using Gaussian 03 program package [21]. The geometries were full optimized using the hybrid B3LYP functional in conjunction with the 6-31+G(d,p) basis set [22]. The harmonic frequencies were calculated at the same level to identify the minima and transation state (TS). The zero-point energies (ZPE) corrections were also obtained from the frequency calculations. Furthermore, the photoionization and dissociation were studied at the B3P86/6-31++G (d, p) level. All the theoretical energies used in this work are electronic energies with ZPE correction. The AE of ionic fragment is defined as *E*_AE_ = *E*_max_ − *E*_0_, in which *E*_max_ refers to the highest energy barrier involved in the formation pathway of corresponding ionic fragment and *E*_0_ is the absolute energy of neutral molecular [23]. Natural bond orbital (NBO) analysis was carried out to characterize the bonds and interactions inside some important species [24].

## Results and discussion

### Photoionization mass spectra

**Fig. 1:**
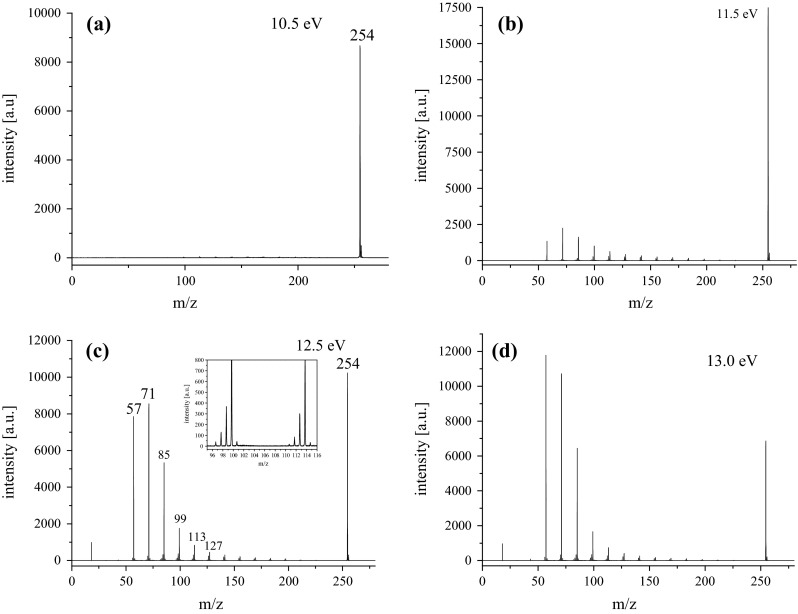
Photoionization mass spectra of octadecane at photon energies of **a** 10.5 eV, **b** 11.5 eV, **c** 12.5 eV, and **d** 13.0 eV

Figure 1 shows the photoionization mass spectra of octadecane at different photon energies. At low photon energy (10.5 eV), the molecular ion at *m*/*z* 254 was detected by near-threshold single-photon ionization (SPI). The fragment ions are negligible, accounting for a soft ionization technique [25–27]. When the photon energy increases to 11.5 eV, fragment ions are formed gradually. At the photon energies of 12.5 and 13 eV, the intensity of relevant fragment ions increases substantially. As shown in Fig. 1, groups of hydrocarbons in fragment ions are clearly observed, and each group has about 2–3 strong ion peaks. The mass difference between two adjacent groups is 14 *m*/*z* units, namely, CH_2_ group, while two adjacent ion peaks in each group have a mass difference of 1 *m*/*z*. These peaks can be mainly attributed to two classes of hydrocarbons: radical hydrocarbon ions (C_*n*_H_2*n*+1_^+^) and alkene ions (C_*n*_H_2*n*_^+^). For example, at the photon energy of 12.5 eV, the main fragment ions are radical ions C_*n*_H_2*n*+1_^+^ (*n* = 4–11) and alkene ions C_*n*_H_2*n*_^+^ (*n* = 5–10). The intensity of peaks follows the order of *m*/*z* 71 > 57 > 85 > 99 > 113. Correspondingly, the fragment ions could be assigned to C_5_H_11_^+^, C_4_H_9_^+^, C_6_H_13_^+^, C_7_H_15_^+^, and C_8_H_17_^+^, respectively. At the left of each radical ion, there is a weak distribution of C_*n*_H_2*n*_^+^ and C_*n*_H_2*n*−1_^+^ ions. Similar results have been reported in the electron-impact (EI) mass spectrum of octadecane at 70 eV [28].

### Photoionization efficiency spectra

**Fig. 2:**
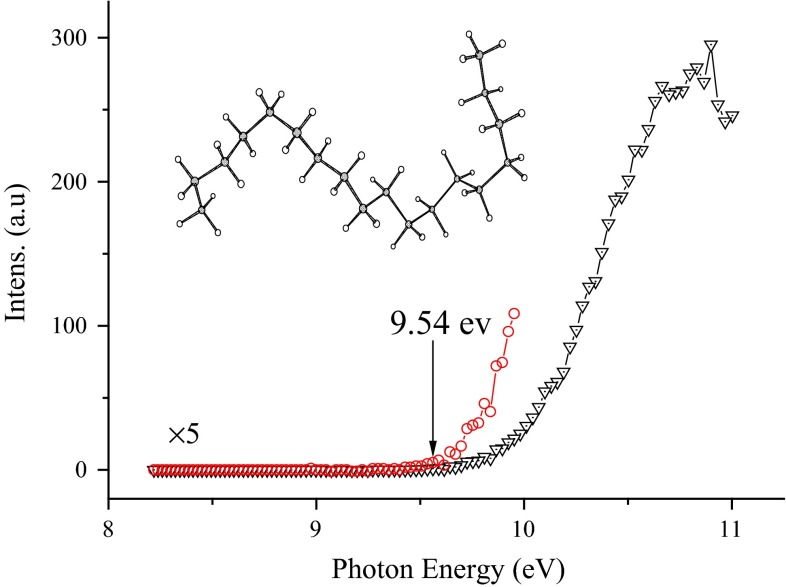
PIE spectrum of molecular ion

The IE value can be measured by scanning PIE spectra, which are obtained by consecutively altering VUV photon energy. The neutral plume of octadecane was generated in the IR laser desorption process. Thus, the hot-band effect will result in a thermal tail in PIE of the molecular ion, which may make it difficult to accurately determine the onset threshold. In addition, weak Franck–Condon factor near the ionization threshold causes a not-obvious onset. Some methods have been employed to determine the ionization threshold [29–31]. In this work, it is assumed that the thermal tail near ionization threshold is dominantly affected by thermal energy from laser heating. The PIE spectrum of octadecane is shown in Fig. 2. It can be found that the IE of octadecane is 9.54 ± 0.05 eV based on the first discernible onset. The calculated adiabatic IE value of octadecane is 9.46 eV by the B3P86/6-31++G(d,p)//B3LYP/6-31+G(d,p) method, according well with the experimental value.

### Fragment ions

The formation of fragment ions has two main pathways. One is direct cleavage of C–C bond to generate both neutral and ionic radicals C_*n*_H_2*n*+1_^+^ (*n* = 4–11); The other occurs via a β-H shift forming alkene ions C_*n*_H_2*n*_^+^ (*n* = 5–10) and alkanes.

**Fig. 3:**
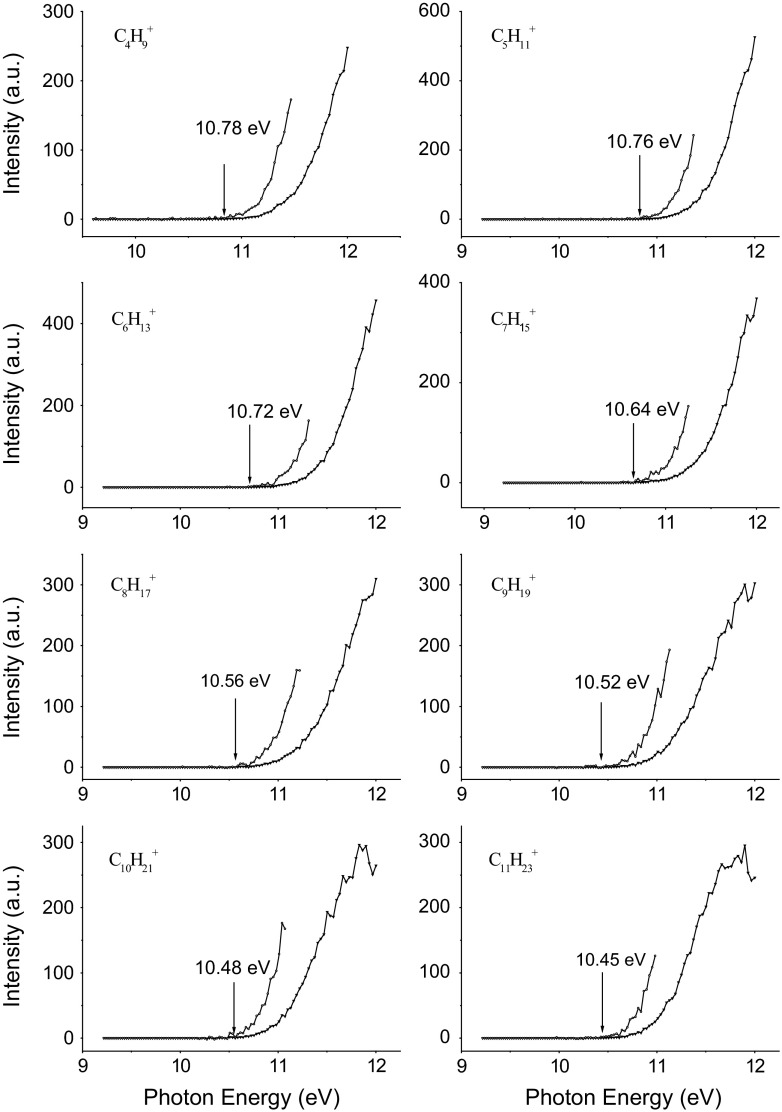
PIE spectra of C_*n*_H_2*n*+1_^+^ radical ions

Figure 3 displays photoionization efficiency spectra of main radical ions C_*n*_H_2*n*+1_^+^ (*n* = 4–11). The AEs of C_4_H_9_^+^, C_5_H_11_^+^, C_6_H_13_^+^, C_7_H_15_^+^, C_8_H_17_^+^, C_9_H_19_^+^, C_10_H_21_^+^, and C_11_H_23_^+^ are 10.78, 10.76, 10.72, 10.64, 10.56, 10.52, 10.48, and 10.45 eV, respectively, indicating a trend of decrease of AEs with the increase of *m*/*z*. The dissociation energies of octadecane were also calculated theoretically (Table 1). They are calculated to be 11.02 eV for C_4_H_9_^+^ + C_14_H_29_, 10.84 eV for C_5_H_11_^+^ + C_13_H_27_, 10.81 eV for C_6_H_13_^+^ + C_12_H_25_, 10.80 eV for C_7_H_15_^+^ + C_11_H_23_, 10.78 eV for C_8_H_17_^+^ + C_10_H_21_, 10.77 eV for C_9_H_19_^+^ + C_9_H_19_, 10.68 eV for C_10_H_21_^+^ + C_8_H_17_, 10.66 eV for C_11_H_23_^+^ + C_7_H_15_ with respect to neutral C_18_H_38_, which are a little larger than the experimental values. The bond order of octadecane ion was obtained on the basis of Wiberg bond index matrix in the NAO basis. It is calculated to be 1.0244, 0.8804, 0.0064, 0.005, 0.0047, 0.0043, 0.0034, 0.0029, and 0.0019 for C^1^–C^2^, C^2^–C^3^, C^3^–C^4^, C^4^–C^5^, C^5^–C^6^, C^6^–C^7^, C^7^–C^8^, C^8^–C^9^, and C^9^–C^10^ (the number of C atom starts from one end of alkyl chain), respectively, indicating that the corresponding strength of C–C bond decreases continuously, which is agreeing with the results of AEs.

**Table 1:** The calculated and experimental energies of products and relevant transition states with respect to neutral octadecane (in eV)

Formula	Calcd	Expt (AE)
C_4_H_9_^+^ + C_14_H_29_	11.02	10.78
C_5_H_11_^+^ + C_13_H_27_	10.84	10.76
C_6_H_13_^+^ + C_12_H_25_	10.81	10.72
C_7_H_15_^+^ + C_11_H_23_	10.80	10.64
C_8_H_17_^+^ + C_10_H_21_	10.78	10.56
C_9_H_19_^+^ + C_9_H_19_	10.77	10.52
C_10_H_21_^+^ + C_8_H_17_	10.68	10.48
C_11_H_23_^+^ + C_7_H_15_	10.66	10.45
C_5_H_10_^+^ + C_13_H_28_	10.28	–
C_6_H_12_^+^ + C_12_H_26_	10.23	–
C_7_H_14_^+^ + C_11_H_24_	10.20	–
C_8_H_16_^+^ + C_10_H_22_	10.18	–
C_9_H_18_^+^ + C_9_H_20_	10.17	–
C_10_H_20_^+^ + C_8_H_18_	10.17	–
TS1	10.82	10.56
TS2	10.75	10.52
TS3	10.70	10.48
TS4	10.68	10.45
TS5	10.64	10.43
TS6	10.58	10.42

**Fig. 4:**
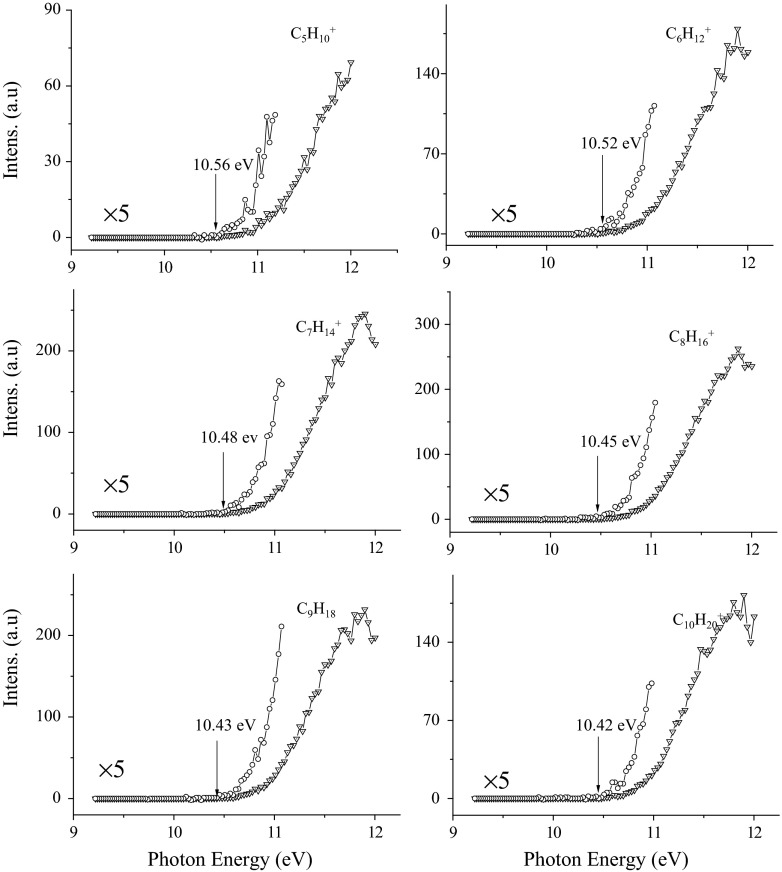
PIE spectra of C_*n*_H_2*n*_^+^ alkene ions

**Fig. 5:**
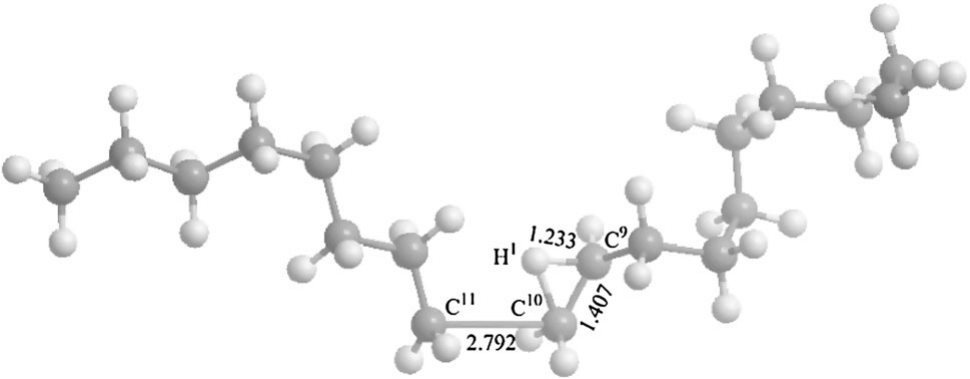
Geometry and selected structural parameters (in Å) optimized at the B3LYP//6-31+G(d,p) level for TS6

The main alkene ions include C_5_H_10_^+^, C_6_H_12_^+^, C_7_H_14_^+^, C_8_H_16_^+^, C_9_H_18_^+^, and C_10_H_20_^+^. The corresponding AEs are found to be 10.56, 10.52, 10.48, 10.45, 10.43, and 10.42 eV, respectively, obtained by the PIE spectra in Fig. 4. Different with direct dissociation into C_*n*_H_2*n*+1_^+^ ions, alkene ions (C_*n*_H_2*n*_^+^) are formed through a β-H shift transition state (TS). That is, octadecane needs to overcome transition state TS1, TS2, TS3, TS4, TS5, and TS6 to form C_5_H_10_^+^ + C_13_H_28_ (calcd 10.28 eV), C_6_H_12_^+^ + C_12_H_26_ (10.23 eV), C_7_H_14_^+^ + C_11_H_24_ (10.20 eV), C_8_H_16_^+^ + C_10_H_22_ (10.18 eV), C_9_H_18_^+^ + C_9_H_20_ (10.17 eV), and C_10_H_20_^+^ + C_8_H_18_ (10.17 eV), respectively. In this process, one C–C bond of octadecane is broken, and then with the help of the bend of C–C skeleton, a hydrogen atom from the β-carbon atom migrates to the other radical carbon, forming a neutral alkane and an alkene ion. For example, in transition state TS6 (Fig. 5), the C^10^–C^11^ bond is calculated to be 2.792 Å, suggesting that it has been broken. The C^9^–H^1^ bond in TS6 is elongated from 1.096 Å in octadecane to 1.233 Å, while the C^9^–C^10^ bond is shorten from 1.515 to 1.407 Å. With respect to neutral octadecane, the energy barrier is calculated to be 10.82 eV for TS1, 10.75 eV for TS2, 10.70 eV for TS3, 10.68 eV for TS4, 10.64 eV for TS5, 10.58 eV for TS6, suggesting that the β-H shift process is the rate-determining step for formation of alkene ions. Although the AEs of alkene ions are less than those of radical ions, formation alkene ions is less kinetically favorable, because it experiences a complex H shift process compared with a simple direct dissociation into radical ions. All these are according with the experimental results.

## Conclusion

The photoionization and fragmentation of octadecane have been investigated with IRLD/VUV PIMS and theoretical calculations. The ionization energy of octadecane was measured to be 9.54 ± 0.05 eV and calculated to be 9.46 eV. The main fragment ions were assigned to radical ions (C_*n*_H_2*n*+1_^+^, *n* = 4–11) and alkene ions (C_*n*_H_2*n*_^+^, *n* = 5–10). The AEs of fragment ions were obtained by measuring the photoionization efficiency spectrum. The AE values of both C_*n*_H_2*n*+1_^+^ and C_*n*_H_2n_^+^ decrease with the increase of the number of C atom. The radical ions C_*n*_H_2*n*+1_^+^ are formed through a direct cleavage of C–C bond in octadecane, while yielding alkene ions C_*n*_H_2*n*_^+^ needs to experience a β-H shift process. This work could be considered as an approach of a combination of IRLD/VUV PIMS and theoretical calculations to research of petroleum.
